# Association of eConsult Implementation With Access to Specialist Care in a Large Urban Safety-Net System

**DOI:** 10.1001/jamahealthforum.2021.0456

**Published:** 2021-05-21

**Authors:** Marema Gaye, Ateev Mehrotra, Hannah Byrnes-Enoch, Dave Chokshi, Andrew Wallach, Laura Rodriguez, Michael L. Barnett

**Affiliations:** 1 Department of Health Policy and Management, Harvard T.H. Chan School of Public Health, Boston, Massachusetts; 2 Department of Health Care Policy, Harvard Medical School, Boston, Massachusetts; 3 New York City Department of Health and Mental Hygiene, New York, New York; 4 Department of Population Health, New York University Grossman School of Medicine, New York, New York; 5 Department of Medicine, New York University Grossman School of Medicine, New York, New York; 6 Office of Ambulatory Care, New York City Health + Hospitals, New York, New York; 7 Division of General Internal Medicine and Primary Care, Department of Medicine, Brigham and Women’s Hospital, Boston, Massachusetts

## Abstract

**Importance:**

Accessing specialty care continues to be a persistent problem for patients who use safety-net health systems. To address this access barrier, hospital systems have begun to implement electronic referral systems using eConsults, which allow clinicians to submit referral requests to specialty clinics electronically and enable specialty reviewers to resolve referrals, if appropriate, through electronic dialogue without an in-person visit.

**Objective:**

Measure the effect of implementing an eConsult program on access to specialty care.

**Design, Setting, and Participants:**

Using an interrupted time series design with data from 2016 to 2020, this study analyzed 50 260 referral requests submitted during the year before and the year after eConsult implementation at 19 New York City Health + Hospitals (NYC H+H) specialty clinics that spanned 7 NYC H+H hospital facilities and 6 unique specialties.

**Exposures:**

Referral request was submitted to a specialty clinic in the year following eConsult implementation.

**Main Outcomes and Measures:**

Main outcomes included the fraction of referral requests resolved without an in-person visit following eConsult implementation; and, among requests triaged to have an in-person visit, the fraction of referrals with a successfully scheduled appointment, mean wait time to a specialty appointment, and the fraction of referral requests with a completed specialty visit. Changes associated with eConsult implementation were estimated using multivariate linear regression adjusting for patient age, gender, and specialty clinic fixed effects.

**Results:**

Across 19 NYC H+H specialty clinics, 26 731 referral requests were submitted in the year before and 23 529 referrals were submitted in the year after eConsult implementation. Following eConsult implementation, 13% of all requests were resolved electronically. Among requests requiring a follow-up visit, the fraction with an appointment successfully scheduled increased by 15.8%, from 66.5% to 82.3% (*P* < .001). The mean time to an appointment decreased from 61.0 days pre-eConsult to 54.1 days post-eConsult, an adjusted 8.2-day shorter wait time (or 13.3% reduction) following eConsult adoption (*P* < .001). The percentage of referrals with a completed follow-up visit with a specialist within 90 days of the request did not change (38.4% vs 37.9%, *P* = .07). Changes in outcomes were mitigated during months when most clinics underwent an electronic health record transition after implementing eConsult.

**Conclusions and Relevance:**

In this quality improvement study, implementation of eConsults at a large multi-specialty safety-net system was associated with improvements in appointment scheduling rates and wait times. Despite an additional electronic health record transition, eConsults are a promising health care delivery tool for increasing access to specialty care.

## Key Points

**Question:**

What was the association of implementation of an eConsult program with access to specialty care in a large safety-net hospital system in the US?

**Findings:**

In this study, 13% of submitted specialty referral requests were resolved electronically and, among requests requiring an in-person visit, appointment scheduling rates increased and wait times to an appointment decreased following eConsult implementation, while visit rates were unchanged. These improvements were mitigated during a hospital-level electronic health record transition.

**Meaning:**

Wide-scale implementation of an eConsult program was associated with reduced wait times for patients in an urban safety-net health system with specialty care needs.

## Teaser

This quality improvement study assesses the association of implementation of an eConsult program with access to specialty care in a safety-net system.

## Introduction

Access to specialty care continues to be a problem for urban underserved populations in the United States. Demand for specialty care is high, with as many as 25% of visits at community health centers resulting in referrals for specialty care,^1^ yet Medicaid enrollees have difficulty obtaining specialty appointments.^2^ In a survey of community health center directors,^3^ 85% reported that their uninsured patients had difficulty accessing new specialty patient visits. This results in many patients not receiving specialty care—only half of uninsured patients reported actually seeing a specialist when recommended to do so.^4^ Barriers to access include long wait times, specialists not accepting new publicly insured or uninsured patients, communication gaps between primary care physicians and specialists, and poor sharing of information.^1,3,5,6^ New models of care that can bridge these gaps are sorely needed to achieve equitable access to specialty care for patients in a safety-net system.

These problems were mirrored in the largest safety-net system in the United States, New York City Health + Hospitals (NYC H+H), estimated to have provided more than 2 million specialty visits in 2015.^7^ Historically, NYC H+H had a fragmented electronic medical record system and no standardized process for ambulatory specialty referrals. In 2016, expansion of an ongoing primary care patient access initiative led to improved data collection for specialty wait times, which demonstrated lengthy wait times and notable variation within and across specialties. This led to NYC H+H ambulatory care clinics prioritizing improvement of communication between primary care and specialty clinicians and standardization of referral workflows. The NYC H+H system began the process of implementing an electronic referral system called eConsult across its hospital facilities and specialty clinics. Using the eConsult workflow, referring clinicians (who were usually, but not always, primary care physicians) submit all referral requests (in contrast to an optional eConsult system) to a particular specialty through the electronic health record (EHR). Each request includes a patient’s clinical background and information on a patient’s health concern that would typically require input from a specialist. Specialty reviewers triage eConsult referral requests to either be scheduled for an appointment or engage in an electronic dialogue with the referring clinician, which can resolve the request without a patient needing a follow-up visit.

Prior literature has found that a substantial fraction of referral requests can be resolved without a patient requiring a face-to-face visit following eConsult implementation,^8,9,10,11,12,13^ and that a higher fraction of patients are able to have appointments scheduled successfully^11,13^ following eConsult implementation. However, prior evidence on eConsult systems’ effects is largely limited in scope (eg, single-specialty clinic in a particular system), or focuses on eConsult programs that do not function as the default referral system for all specialty requests (eg, are optional) as implemented in NYC H+H and other safety-net health systems.^9,14,15,16,17,18,19,20,21^

To our knowledge, there has been no prior evaluation of a systemwide, multispecialty eConsult implementation that uses data before and after eConsult implementation to evaluate temporal trends in wait times and other outcomes. To address this evidence gap, we examined referral requests before and after eConsult adoption at several NYC H+H specialty clinics to understand the associations of the program with access to specialty care for patients within the NYC H+H system.

## Methods

### The eConsult Program at NYC H+H

The NYC H+H system began rolling out its eConsult platform through a pilot initiative beginning in August 2016. After a successful pilot test of eConsult at 3 hospital facilities, specialty clinics could voluntarily adopt the platform beginning in 2017, and by the end of 2019, 158 specialty clinics across 12 NYC H+H facilities had implemented the eConsult platform. After a specialty clinic adopted the eConsult platform, all referrals from other outpatient clinicians to their department were required to flow through eConsult. Starting in 2020, NYC H+H made eConsult implementation a requirement for all of its ambulatory specialty clinics, with limited exceptions identified for specialties that were considered part of primary care (eg, obstetrics/gynecology), were procedure based (eg, colonoscopy, radiology), or were otherwise inappropriate for an electronic referral workflow (eg, certain behavioral health programs).

Each specialty clinic director was responsible for ensuring that all eConsult requests were reviewed within 72 hours of their submission by a designated specialty reviewer. If the reviewer determined that a specialty visit was necessary based on the referring clinician’s request, the request was forwarded to staff at the specialty clinic, who began the process of appointment booking with the patient. For a substantial fraction of eConsults where clinical management may have been possible without a specialty visit, the reviewer engaged in an electronic dialogue with the primary care professional that could resolve the clinical request without requiring a visit with a specialist.

The eConsult workflow was built into each specialty clinic’s EHR system. In addition to rolling out the eConsult program, NYC H+H transitioned its hospital facilities and specialty clinics from using the QuadraMed EHR system to using the Epic EHR system in a staggered rollout beginning in 2016. Since the eConsult workflow was built into the EHR system, several specialty clinics both changed EHR systems and integrated the eConsult platform within months of each other.

This study was determined to be not human participant research and therefore was exempt from review by the Biomedical Research Alliance of New York Institutional Review Board and Harvard T.H. Chan School of Public Health. The study followed the Standards for Quality Improvement Reporting Excellence (SQUIRE) reporting guidelines.

### Data Sources

To analyze the association of the eConsult program with access to specialty care, we used a database of all referral requests submitted to 19 NYC H+H specialty clinics through the QuadraMed and Epic EHR systems between January 2016 and February 2020. We linked each request to all related outpatient specialty encounters (eg, appointments scheduled and follow-up visits, see eMethods in the Supplement). The database included referrals across 19 separate clinical departments representing 6 separate specialties (cardiology, endocrinology, gastroenterology, neurology, nephrology, and urology) at 7 NYC H+H hospital facilities (Bellevue, Elmhurst, Jacobi, Lincoln, Metropolitan, North Central Bronx, and Woodhull). For each referral request, the database captured the date of the referral request, whether an appointment was scheduled following the referral request, and whether a follow-up visit occurred related to the referral. The database also recorded the triage decision (eg, schedule appointment vs respond with electronic message) made by the specialty reviewer for each request submitted after eConsult adoption. For each specialty clinic, we also obtained information on the date on which the eConsult platform went live at the specialty clinic (eConsult start date) and the date on which the specialty clinic began using the Epic EHR system (Epic start date).

### eConsult and Referral Study Sample

For each referral request, we limited our sample to encounters with a scheduling action (ie, an action by administrative staff to schedule an appointment) within 30 days of the referral request. Among the remaining encounters, we identified the first triage decision, date of the first appointment scheduled for the matching specialty clinic (which may have been different than the date of a completed visit), and the date of the first completed office visit occurring within 365 days of the referral request to the matching specialty clinic for each patient.

We then limited our sample to referral requests from each specialty clinic that occurred between the period 12 months before and 12 months after eConsult adoption at the specialty clinic. We also further excluded referral requests based on the quality of data recorded for those requests (eMethods in the Supplement). Multiple referrals for the same patient over time were included in the analysis. A sensitivity analysis excluding 9610 repeat referrals only trivially affected the results reported below (eTable 1 in the Supplement).

### Outcomes

We first examined the monthly volume of eConsult requests across the NYC H+H system. We classified the outcomes of each referral request as either “follow-up visit to specialist scheduled” if the specialty reviewer decided the patient needed a specialist visit, or “resolved without a visit” if the reviewer could resolve an eConsult request without an in-person visit through a dialogue with the referring clinician. We measured the percentages of eConsult requests categorized as resolved without a visit across the whole study sample and by specialty clinic.

Among referral requests triaged to have a follow-up visit to specialist scheduled, we measured the percentage of referrals that resulted in an appointment being scheduled and then, among referral requests with a scheduled appointment, we calculated the wait time in days between the scheduled appointment and the date of the original eConsult request. We also measured the percentage of referrals linked to a follow-up visit within 90 days of the date the request was submitted.

### Patient and eConsult Covariates

Each referral request was assigned to the specialty clinic that received the request. We captured the age and gender of each referred patient. We identified whether the referral request occurred in the 12-month period before eConsult adoption (ie, pre-eConsult) or the 12-month period after eConsult adoption (ie, post-eConsult). We also identified when the request occurred by month relative to eConsult adoption at the specialty clinic in 30-day increments and assigned each request to a relative month (see eMethods in the Supplement).

### Statistical Analysis

We compared the characteristics of patients with referrals and unadjusted rates of outcomes in the pre-eConsult and post-eConsult periods with bivariate statistical tests. We then conducted an interrupted time series analysis of the eConsult program using the database of eConsult requests and linked encounters. We conducted adjusted analyses with individual referral-level multivariate linear regression and the margins command in Stata (v. 15, StataCorp) to calculate adjusted proportions and means for each outcome over time. For the adjusted results, our key quantity of interest was an indicator for whether a referral request was submitted to a clinic after eConsult was adopted at the clinic (ie, the post-eConsult period), which provides an estimate of the average change in the particular outcome in the post-eConsult period compared with the pre-eConsult period. All regression models included specialty clinic fixed effects to control for observed and unobserved characteristics of each specialty clinic. We also used clustered standard errors at the specialty clinic level to account for correlation of patient outcomes (eg, wait time) within the clinic. Although patient referrals are also nested within primary care physicians and specialist reviewers, the specialty clinic is the most influential level for determining outcomes related to appointment scheduling and wait times. All *P* values were 2-tailed, and *P* < .05 was considered statistically significant.

## Results

The eConsult program was voluntarily rolled out at the 19 specialty clinics in the study sample between 2017 and 2019. During the period 12 months before and 12 months after eConsult adoption, 50 260 referral requests were submitted to these specialty clinics. Characteristics of patients for whom the request was submitted were similar in the pre-eConsult and post-eConsult period: the mean (SD) age of patients with a referral request was 55.5 (16.4) years pre-eConsult and 56.8 (16.1) years post-eConsult (*P* < .001), and 52.4% of patients in the pre-eConsult period and 52.6% of patients in the post-eConsult period were female (*P* = .76) (eTable 2 in the Supplement).

**Figure 1.**
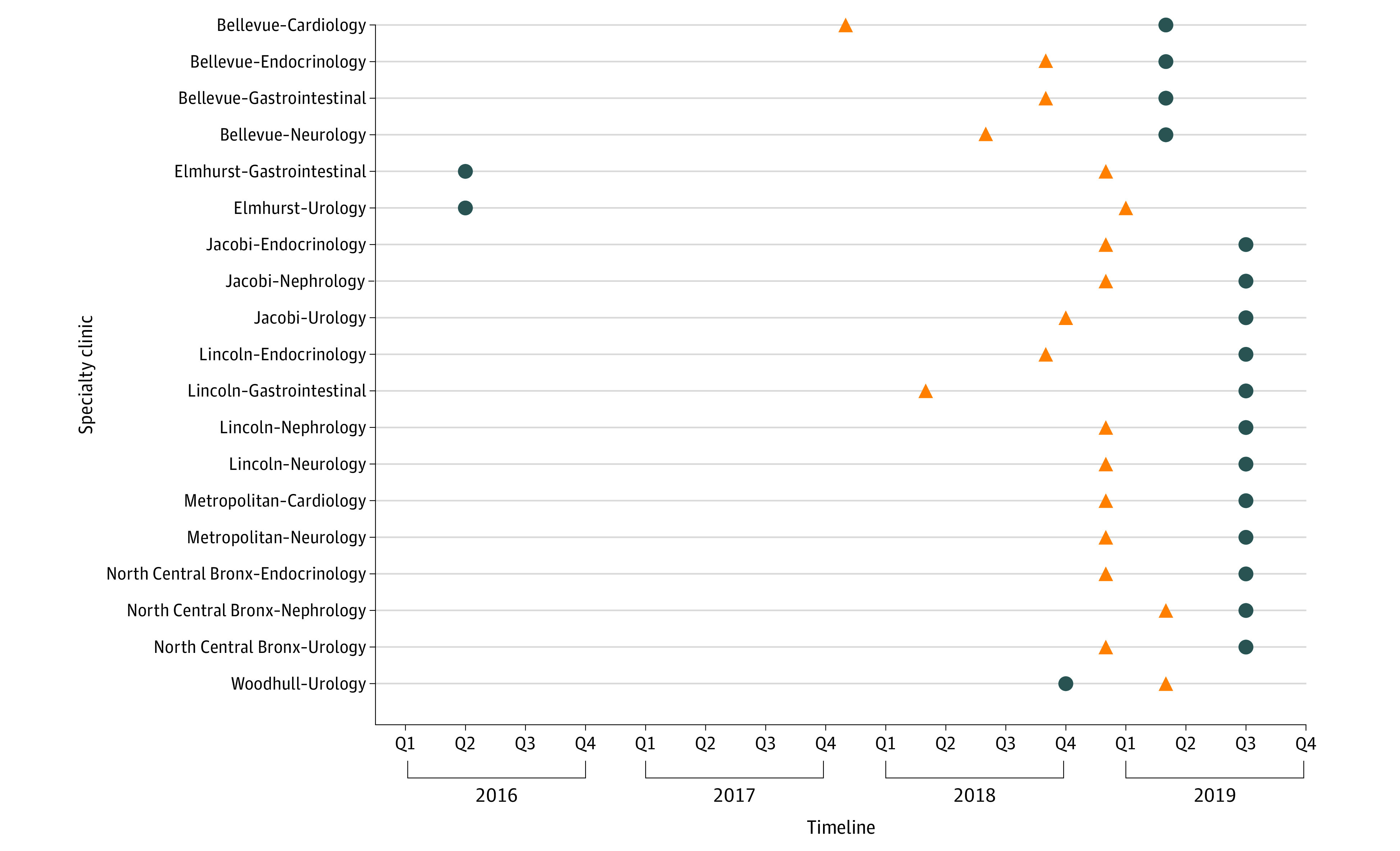
Timeline of eConsult Adoption and Electronic Health Record Transitions at NYC Health + Hospitals Specialty Clinics The triangles represent eConsult transition; the dots, electronic health record transition.

The timing of the EHR transition from QuadraMed to Epic relative to eConsult adoption varied across the specialty clinics in the sample (Figure 1). Of the 19 specialty clinics, 3 clinics transitioned EHR systems before adopting eConsult. The remaining 16 specialty clinics transitioned EHR systems after adopting eConsult, and 10 of these clinics (more than half of the specialty clinic sample) transitioned EHR systems in the seventh or eighth month of eConsult implementation.

The monthly volume of referrals across all clinics decreased from an average of 2228 referrals per month before eConsult to 1961 referrals per month post-eConsult (eFigure 1 in the Supplement). The average monthly volume of referrals at each specialty clinic varied widely from 28 to 305 referrals in the pre-eConsult period and from 24 to 304 referrals in the post-eConsult period (eTable 3 in the Supplement).

**Figure 2.**
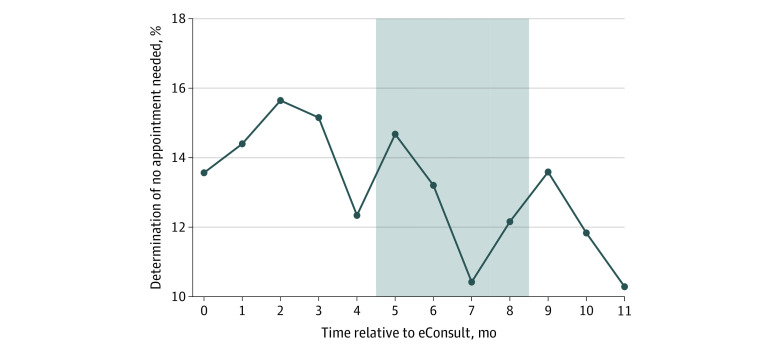
Proportion of eConsults Where It Was Determined No Appointment Needed The gray shaded region indicates months where 10 of 19 facilities transitioned electronic health record systems. Month 0 is the first 30 days of eConsult.

In the 12-month period following eConsult implementation, 3 074 out of 23 529 (13%) of referral requests across all 19 specialty clinics were resolved without requiring a follow-up specialty appointment for the patient. As shown in Figure 2, this rate varied in the months that followed eConsult adoption from 10% (219 out of 2 130 referral requests in the twelfth month post-eConsult) to 16% (298 out of 1 905 referral requests in the third month post-eConsult). The percentage of referral requests that were resolved without a visit varied considerably between specialty clinics, ranging from 2.4% to 42.3% (eTable 4 in the Supplement), with substantial variation in the monthly rate of resolved referrals for each specialty clinic (eFigure 2 in the Supplement).

**Figure 3.**
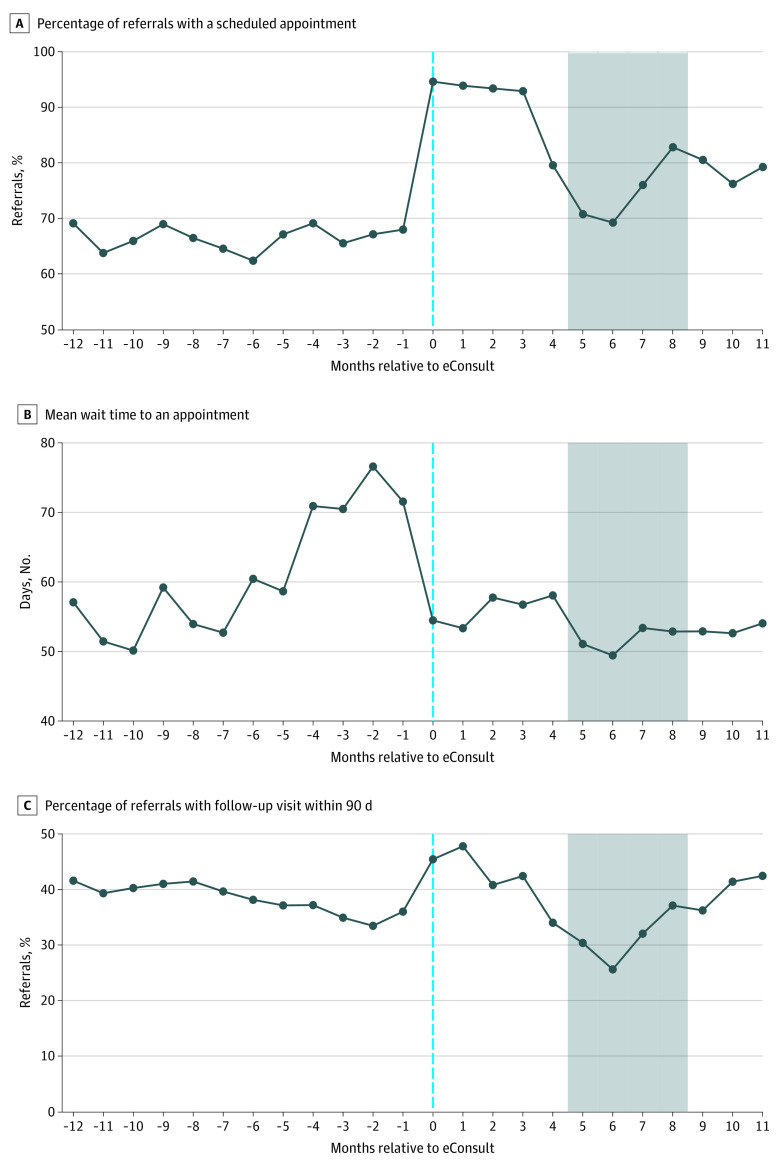
Outcomes Among Referrals Triaged to Have a Follow-up Visit Scheduled, by Month Relative to eConsult Adoption The gray-shaded region indicates months where 10 of 19 facilities transitioned electronic health record systems. Month 0 is the first 30 days of eConsult. A, Percentage of referrals with a scheduled appointment. B, Referrals without an appointment scheduled (n = 12 574) are excluded. C, Referrals resolved that occurred within 90 days of the end of the study period (n = 726) are excluded.

Among the remaining referral requests that were triaged to have a follow-up specialty visit scheduled, the percentage of referrals with an appointment scheduled increased from 66.5% in the pre-eConsult period to 82.3% in the post-eConsult period (*P* < .001, Table). In the first 4 months following eConsult adoption, the scheduling rate initially increased to nearly 95%, then decreased over the next 4 months during the period in which multiple specialties were undergoing EHR transitions (Figure 3). The scheduling rate remained steady near 80% in the last 3 months of the time series. Of the 19 specialty clinics, 16 experienced increases in scheduling rates, ranging from 2.4% to 49.2% (eTable 5 in the Supplement).

**Table.** Outcomes Among Referrals Triaged to Have a Follow-up Visit Scheduled, Pre-eConsult vs Post-eConsult Adoption

Outcomes	eConsult, No. (%)		Adjusted^a^		Relative change, %
	Before	After	Difference	*P* value	
Referrals with an appointment scheduled^b^	17 781 (66.5)	16 831 (82.3)	+15.8%	<.001	+23.8
Wait time to appointment, mean (SD), d^c^	60.1 (59.2)	54.1 (34.8)	−8.2	<.001	−13.3
Referrals with visit occurring within 90 d^d^	10 267 (38.4)	7472 (37.9)	−0.8%	.07	−2.1

^a^ Adjusted differences and *P* values were calculated using a linear regression and the margins function in Stata (v.15). The regression included an indicator for whether the referral occurred in the 12-month post-eConsult adoption period at the specialty clinic the patient was being referred to, with specialty clinic fixed effects.

^b^ Referrals that resolved without a face-to-face visit (n = 3074) are excluded.

^c^ Referrals that resolved without a face-to-face visit (n = 3074) or without an appointment scheduled (n = 12 574) are excluded.

^d^ Referrals that resolved without a face-to-face visit (n = 3074) or that occurred within 90 days of the end of the study period (n = 726) are excluded.

Among referral requests with an appointment scheduled, the mean time to an appointment decreased from 61.0 days pre-eConsult to 54.1 days post-eConsult (Table), an adjusted 8.2-day shorter wait time (or 13.3% reduction) to specialty appointments following eConsult adoption (*P* < .001). After increasing in the months prior to eConsult adoption, following eConsult adoption, wait times to an appointment initially decreased from 70 days in the month before eConsult adoption to 55 days in the first month of eConsult. The difference in mean time to an appointment also varied by specialty clinic. Overall, 13 of the 19 specialty clinics experienced decreases in wait time to an appointment, ranging from a 3.1-day shorter wait time to a 57.2-day shorter wait time (eTable 5 in the Supplement).

The percentage of referrals with a follow-up visit with a specialist within 90 days of the request did not change after eConsult adoption overall (38.4% vs 37.9%, *P* = .07). However, this average result obscures some nonlinear trends post-eConsult (Figure 3). Follow-up visit rates increased to 47.8% by the second month post-eConsult, before decreasing over the following 5 months to 25.6% during the EHR transition period, then increasing over the remaining 5 months to 42.5% in the last month of the time series. A total of 11 of the 19 specialty clinics experienced increases in completed 90-day follow-up visit rates following eConsult implementation, ranging from 0.6% to 21.6% (eTable 5 in the Supplement).

## Discussion

In the first 12 months after implementation of eConsult at NYC H+H, the program made significant progress on several of its goals. The average wait times for specialty appointments fell by 8.2 days, a reduction of 13.3%, while the number of referrals with a successfully scheduled appointment increased by 15.8%. There was no significant change in the proportion of referrals that resulted in a completed follow-up specialty visit within 90 days. This new evidence is valuable because, aside from a small, single-system randomized clinical trial for cardiology eConsults,^8^ much of the existing data on eConsults comes from single-facility, single-specialty evaluations with limited control period data. To our knowledge, this evaluation is the first study of a multispecialty eConsult implementation across a health system that compares care before and after the implementation.

The present study findings add to recent evidence that electronic referral systems such as eConsult are a promising tool for improving access to and the delivery of specialty care for patients using safety-net systems. Resolving requests through eConsult potentially freed up scheduling space in the early months of the program and enabled reviewing health care professionals to prioritize appointment scheduling for patients who may have had more urgent health concerns. Additionally, despite a larger fraction of the remaining referrals requiring a follow-up visit having an appointment successfully scheduled, average wait times improved. Because of this, the program may have allowed patients with more urgent needs to be seen sooner. This is consistent with prior evidence showing that some health care professionals often used the platform to resolve minor health concerns, have administrative or clinical questions answered, and provide previsit evaluations that, in the absence of eConsult, would have normally required that a patient have an in-person appointment.^10,14,18,22^ Another benefit is that specialty reviewers triaging the urgency of eConsults can rush urgent referral requests to earlier appointments and push elective visits to later appointment dates.

While scheduling rates and average wait times improved across the specialty clinics, there is clearly much room for improvement. The average patient needing a specialty visit had an appointment scheduled for nearly 2 months after the initial referral submission. Furthermore, more than half of patients with a referral still did not complete an in-person appointment within 90 days following eConsult implementation, and there was no improvement on this outcome.

Interpretation of these results must consider the wide-scale EHR transition across the NYC H+H system beginning approximately 6 months into many hospitals’ eConsult implementations. The EHR transition was a substantial disruption that both affected how appointments were scheduled and limited clinicians’ capacity to see patients during the time they were receiving training on the transition and for several weeks after transition. In the first 5 to 6 months after eConsult implementation, all 3 of the main study outcomes saw meaningful improvements in the direction we would hypothesize. However, these improvements diminished substantially during the EHR transition period. This limits our ability to project the long-term associations of eConsult in this system, especially because our study period ended right before the rise of the COVID-19 pandemic in New York City. It is still plausible, though not testable with the present data, that in the absence of a more efficient scheduling system such as eConsult, the transition may have caused a worse disruption than we observed in specialty access outcomes.

These results suggest other steps that could be pursued to improve the referral process and access to specialty care. The 13% rate of referrals that resolved without a patient requiring an in-person visit is lower than that found in other health systems.^9,15^ Further improvements to the eConsult program could include implementing more consistent training for specialty reviewers to identify which referral requests may be more appropriately resolved through electronic dialogue rather than a patient needing an in-person visit. Another challenge was the persistently low appointment completion rate despite improved scheduling and wait times. It is difficult for patients to predict their ability to attend an appointment 2 months in the future. There may need to be additional support including patient-centered reminders.^3,23^

### Limitations

The present study has several limitations. First, although we used an interrupted time series design, we lacked a control group to assess whether differences in outcomes between the pre-eConsult and post-eConsult adoption were due to eConsult. Second, we lacked detailed clinical information on patients or individual eConsult requests beyond which specialty clinic the request was sent to, which limited our ability to assess referral appropriateness. Third, we were unable to determine whether an eConsult request was resolved because the referring physician received clinical advice or if additional information was needed before the specialty reviewer could determine whether the request should be triaged to have a specialty appointment. Fourth, because we lacked a control group, we are unable to definitively assess how much the trends we observed deviated from secular trends that may have been occurring systemwide independent of eConsult implementation. In addition, this study focused on a single system’s implementation in New York City, and the results may not be generalizable to other systems across the United States. However, the study analyzed what we believe is the largest implementation of an eConsult system in the country.

## Conclusions

In conclusion, this evaluation adds further evidence on the potential benefit for safety-net health systems struggling with improving specialty care access under resource constraints. Additional efforts aimed at increasing access to specialty care should both consider ways to further improve on wait times to appointments and strategies to increase overall visit rates, such as targeted appointment reminders or other behavioral nudges to improve visit attendance.

## Notes

Supplement. eMethods. eTable 1. Outcomes among first referral requests submitted for a patient to a particular specialty clinic, pre- vs. post-eConsult adoption eTable 2. Patient/Referral-level characteristics eFigure 1. Monthly volume of specialty referral requests eTable 3. Mean monthly volume of referrals pre-eConsult vs. post-eConsult adoption, by specialty clinic eTable 4. Percent of referrals resolved without requiring face-to-face visit, by specialty clinic eFigure 2. Percent of referrals resolved without requiring face-to-face visit per month relative to eConsult adoption, by specialty clinic eTable 5. Difference in outcomes among referrals triaged to have a follow-up visit pre-eConsult vs. post-eConsult adoption, by specialty clinic
