# Supplementary material for: Association of eConsult Implementation With Access to Specialist Care in a Large Urban Safety-Net System
Source: JAMA Health Forum. 2021 May 21;2(5):e210456. doi: 10.1001/jamahealthforum.2021.0456 (PMC8796905; doi:10.1001/jamahealthforum.2021.0456)
Supplement: Supplement. — eMethods. eTable 1. Outcomes among first referral requests submitted for a patient to a particular specialty clinic, pre- vs. post-eConsult adoption eTable 2. Patient/Referral-level characteristics eFigure 1. Monthly volume of specialty referral requests eTable 3. Mean monthly volume of referrals pre-eConsult vs. post-eConsult adoption, by specialty clinic eTable 4. Percent of referrals resolved without requiring face-to-face visit, by specialty clinic eFigure 2. Percent of referrals resolved without requiring face-to-face visit per month relative to eConsult adoption, by specialty clinic eTable 5. Difference in outcomes among referrals triaged to have a follow-up visit pre-eConsult vs. post-eConsult adoption, by specialty clinic [file jamahealthforum-e210456-s001.pdf]

# Supplemental Online Content

Gaye M, Mehrotra A, Byrnes-Enoch H, et al. Association of eConsult implementation with access to specialist care in a large urban safety-net system. *JAMA Health Forum.* 2021;(2)5:e210456. doi:10.1001/jamahealthforum.2021.0456

**eMethods.**
**eTable 1.** Outcomes among first referral requests submitted for a patient to a particular specialty clinic, pre- vs. post-eConsult adoption
**eTable 2.** Patient/Referral-level characteristics
**eFigure 1**. Monthly volume of specialty referral requests
**eTable 3**. Mean monthly volume of referrals pre-eConsult vs. post-eConsult adoption, by specialty clinic
**eTable 4**. Percent of referrals resolved without requiring face-to-face visit, by specialty clinic
**eFigure 2**. Percent of referrals resolved without requiring face-to-face visit per month relative to eConsult adoption, by specialty clinic
**eTable 5**. Difference in outcomes among referrals triaged to have a follow-up visit pre-eConsult vs. post-eConsult adoption, by specialty clinic

This supplemental material has been provided by the authors to give readers additional information about their work.

© 2021 Gaye et al. *JAMA Health Forum.*

**eMethods**

1) **Source of referral request and outpatient encounter data**

We used a database of all referral requests submitted through the QuadraMed and Epic EHR systems across 19 NYC H+H specialty clinics between January 2016 and February 2020.

We linked each referral request to all related outpatient specialty encounters. Outpatient specialty encounters were pulled from both Epic and the legacy patient scheduling system (Soarian) which supported QuadraMed. We linked each outpatient encounter to a referral request if the encounter occurred for the same patient within 365 days from when the referral request was submitted. Only one specialty encounter, the closest in time, was linked for every referral request.

2) **Assignment of relative month to each referral request**

For each referral request, we calculated the number of days between the submission date of the referral request and the eConsult start date at the specialty clinic it was submitted to. Then, we assigned each referral to a "relative month" based on 30-day increments of when they were submitted relative to eConsult implementation. Referral requests submitted within the first 30 days of eConsult (the eConsult start date plus the following 29 days) at a particular specialty clinic were assigned to relative month 0. For referral requests that occurred after relative month 0 of eConsult, referrals that occurred within day 31-60 of eConsult were assigned to relative month 1, referrals that occurred within day 61-90 of eConsult were assigned to relative month 2, and so on. For referral requests that occurred before the eConsult adoption date, we assigned referrals that occurred in the period 30 days before eConsult to relative month -1, and so on.

3) **Referral request sample exclusions**

© 2021 Gaye et al. *JAMA Health Forum.*

For each referral request, we limited our sample to encounters with a scheduling action (i.e. an action by administrative staff to schedule an appointment) within 30 days of the referral request. Among the remaining encounters, we identified the first triage decision, date of the first appointment scheduled for the matching specialty clinic (which may have been different than the date of a completed visit), and the date of the first completed office visit occurring within 365 days of the referral request to the matching specialty clinic for each patient.

We then limited our sample to referral requests from each specialty clinic that occurred between the period 12 months before and 12 months after eConsult adoption at the specialty clinic. We excluded referrals with missing data on the referring physician or target specialty. We also excluded referrals from specialty clinics with a data break during 24-month period of analysis (i.e. missing all data for 1 or more months) and referrals from clinics with less than 24 continuous months of referral data. Finally, based on input from NYC H+H leadership, we excluded referrals from specialty clinics at the Harlem Hospital facility. At this facility, implementation of eConsult and the eConsult workflow substantially differed from other facilities.

© 2021 Gaye et al. *JAMA Health Forum.*

**eTable 1.** Outcomes among first referral requests submitted for a patient to a particular specialty clinic, pre- vs. post-eConsult adoption

| Outcomes | Pre-eConsult | Post-eConsult | Adjusted difference [a] | Adjusted p-value [a] | Relative change |
|---|---|---|---|---|---|
| **Total referrals** | 23,700 | 16,950 | | | |
| **Referrals resolved without a face-to-face visit** | - | 2,354 (13.9%) | | | |
| **Referrals triaged to have a follow-up visit** | 23,700 (100%) | 14,596 (86.1%) | | | |
| *Among referrals triaged to have a follow-up visit* | | | | | |
| **Referrals with an appointment scheduled, n (%)** [b] | 15,751 (66.5%) | 12,001 (82.2%) | +16.2% | p<0.001 | +23.6% |
| **Wait time to appointment in days, mean (SD)** [c] | 61.1 (59.5) | 54.6 (35.6) | -8.1 | p<0.001 | -10.6% |
| **Referrals with visit occurring within 90 days, n (%)** [d] | 9,109 (38.4%) | 5,319 (37.6%) | -0.7% | 0.15 | -2.1% |

[a] Adjusted differences, and p-values were calculated using a linear regression and the margins function in Stata (v.15, College Station, TX). The regression included an indicator for whether the referral occurred in the 12-month post-eConsult adoption period at the specialty clinic the patient was being referred to, with specialty clinic fixed effects.
[b] Referrals resolved without a face-to-face visit (n = 2,354) are excluded.
[c] Referrals resolved without a face-to-face visit (n = 2,354) or without an appointment scheduled (n = 10,544) are excluded.
[d] Referrals resolved without a face-to-face visit (n = 2,354) or that occurred within 90 days of the end of the study period (n = 462) are excluded.

© 2021 Gaye et al. *JAMA Health Forum.*

**eTable 2**. Patient/Referral-level characteristics

| | **Overall** | **Pre-eConsult** | **Post-eConsult** | **p-value[a]** |
|---|---|---|---|---|
| Total referrals | 50,260 | 26,731 | 23,539 | |
| Patient age in years, mean (SD)[b] | 56.1 (16.3) | 55.5 (16.4) | 56.8 (16.1) | p<0.001 |
| Patient gender, n (%)[c] | | | | 0.76 |
| Male | 23,813 (47.4%) | 12,653 (47.3%) | 11,160 (47.4%) | |
| Female | 26,315 (52.4%) | 13,947 (52.2%) | 12,368 (52.6%) | |

[a] T-test was performed on patient age. Chi-squared test was performed on patient gender.
[b] Referrals with missing patient age (n = 131) are excluded.
[c] Referrals with missing patient gender (n = 132) are excluded.
SD = Standard Deviation.

© 2021 Gaye et al. *JAMA Health Forum.*

**eFigure 1**. Monthly volume of specialty referral requests

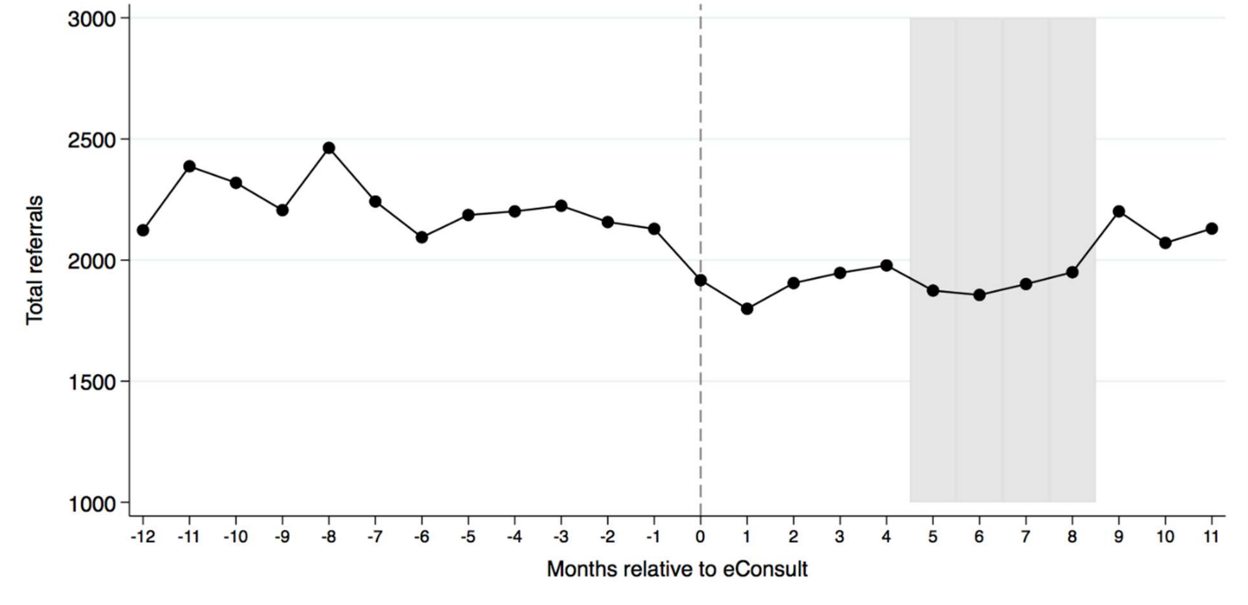


Notes: The gray shaded region indicates months where 10 of 19 facilities transitioned EHR systems. Month 0 is the first 30 days of eConsult.

© 2021 Gaye et al. *JAMA Health Forum.*

**eTable 3**. Mean monthly volume of referrals pre-eConsult vs. post-eConsult adoption, by specialty clinic

| Specialty clinic | Pre-eConsult | Post-eConsult | Difference |
|---|---|---|---|
| Bellevue Cardiology | 160 | 163 | 4 |
| Bellevue Endocrinology | 117 | 117 | 0 |
| Bellevue Gastrointestinal | 305 | 304 | -1 |
| Bellevue Neurology | 164 | 168 | 5 |
| Elmhurst Gastrointestinal | 227 | 197 | -30 |
| Elmhurst Urology | 159 | 159 | 0 |
| Jacobi Endocrinology | 44 | 55 | 11 |
| Jacobi Nephrology | 38 | 38 | -1 |
| Jacobi Urology | 54 | 73 | 19 |
| Lincoln Endocrinology | 74 | 67 | -7 |
| Lincoln Gastrointestinal | 128 | 78 | -49 |
| Lincoln Nephrology | 76 | 50 | -25 |
| Lincoln Neurology | 99 | 84 | -16 |
| Metropolitan Cardiology | 111 | 91 | -20 |
| Metropolitan Neurology | 227 | 130 | -97 |
| North Central Bronx Endocrinology | 33 | 34 | 1 |
| North Central Bronx Nephrology | 28 | 24 | -4 |
| North Central Bronx Urology | 53 | 34 | -19 |
| Woodhull Urology | 130 | 94 | -36 |

© 2021 Gaye et al. *JAMA Health Forum.*

**eTable 4**. Percent of referrals resolved without requiring face-to-face visit, by specialty clinic

| Specialty clinic | Percent of referral requests resolved without a visit |
|---|---|
| Bellevue Cardiology | 2.7% |
| Bellevue Endocrinology | 7.8% |
| Bellevue Gastrointestinal | 13.6% |
| Bellevue Neurology | 6.2% |
| Elmhurst Gastrointestinal | 19.4% |
| Elmhurst Urology | 3.6% |
| Jacobi Endocrinology | 33.9% |
| Jacobi Nephrology | 10.8% |
| Jacobi Urology | 12.4% |
| Lincoln Endocrinology | 15.9% |
| Lincoln Gastrointestinal | 42.3% |
| Lincoln Nephrology | 18.7% |
| Lincoln Neurology | 31.1% |
| Metropolitan Cardiology | 5.9% |
| Metropolitan Neurology | 4.4% |
| North Central Bronx Endocrinology | 17.4% |
| North Central Bronx Nephrology | 2.4% |
| North Central Bronx Urology | 11.7% |
| Woodhull Urology | 15.2% |

© 2021 Gaye et al. *JAMA Health Forum.*

**eFigure 2**. Percent of referrals resolved without requiring face-to-face visit per month relative to eConsult adoption, by specialty clinic

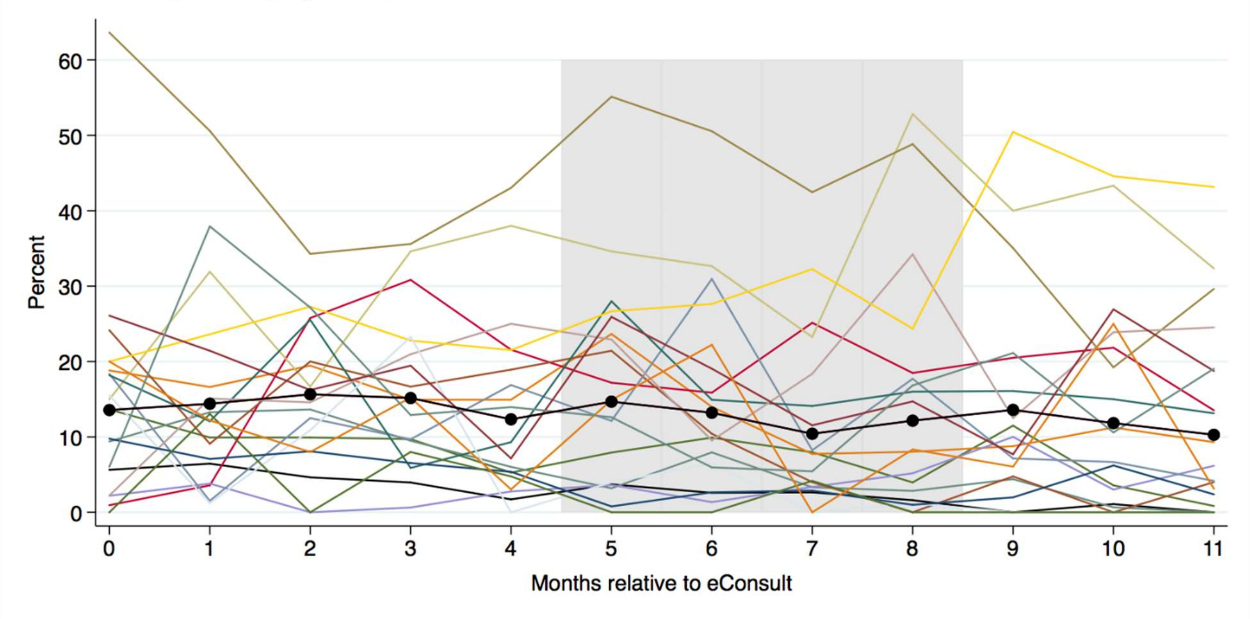


Notes: The gray shaded region indicates months where 10 of 19 facilities transitioned EHR systems. Month 0 is the first 30 days of eConsult.

© 2021 Gaye et al. *JAMA Health Forum.*

**eTable 5**. Difference in outcomes among referrals triaged to have a follow-up visit pre-eConsult vs. post-eConsult adoption, by specialty clinic

| | **Percent of referrals with a scheduled appointment** | | | **Mean wait time to appointment, in days** | | | **Percent of referrals with a follow-up visit within 90 days** | | |
|---|---|---|---|---|---|---|---|---|---|
| Specialty clinic | Pre-eConsult | Post-eConsult | Difference | Pre-eConsult | Post-eConsult | Difference | Pre-eConsult | Post-eConsult | Difference |
| Bellevue Cardiology | 96.8% | 99.2% | 2.4% | 35.8 | 39.0 | 3.3 | 67.0% | 68.6% | 1.6% |
| Bellevue Endocrinology | 48.1% | 97.3% | 49.2% | 66.0 | 62.6 | -3.4 | 35.1% | 40.9% | 5.8% |
| Bellevue Gastrointestinal | 60.1% | 97.8% | 37.7% | 63.2 | 64.3 | 1.2 | 43.6% | 39.7% | -3.9% |
| Bellevue Neurology | 58.0% | 99.0% | 41.0% | 60.6 | 57.5 | -3.1 | 43.2% | 43.8% | 0.6% |
| Elmhurst Gastrointestinal | 50.3% | 42.5% | -7.9% | 137.4 | 80.1 | -57.2 | 12.7% | 18.7% | 6.0% |
| Elmhurst Urology | 63.1% | 50.2% | -12.9% | 117.6 | 70.4 | -47.2 | 15.3% | 21.3% | 6.0% |
| Jacobi Endocrinology | 70.9% | 99.3% | 28.4% | 79.4 | 57.0 | -22.4 | 31.4% | 38.4% | 7.0% |
| Jacobi Nephrology | 78.9% | 98.0% | 19.2% | 62.9 | 40.2 | -22.6 | 42.0% | 48.1% | 6.0% |
| Jacobi Urology | 35.9% | 88.9% | 53.1% | 41.1 | 50.6 | 9.5 | 22.4% | 31.1% | 8.7% |
| Lincoln Endocrinology | 84.9% | 97.6% | 12.8% | 42.4 | 38.3 | -4.1 | 58.0% | 39.1% | -18.9% |
| Lincoln Gastrointestinal | 90.2% | 99.4% | 9.3% | 18.7 | 20.1 | 1.4 | 66.9% | 56.4% | -10.5% |
| Lincoln Nephrology | 89.2% | 90.9% | 1.6% | 45.2 | 41.6 | -3.5 | 60.1% | 40.3% | -19.7% |
| Lincoln Neurology | 89.8% | 99.0% | 9.1% | 20.8 | 25.3 | 4.4 | 66.4% | 54.6% | -11.7% |
| Metropolitan Cardiology | 68.2% | 74.9% | 6.7% | 54.5 | 50.3 | -4.3 | 43.9% | 34.4% | -9.6% |
| Metropolitan Neurology | 53.0% | 48.7% | -4.4% | 64.1 | 58.4 | -5.7 | 22.7% | 19.5% | -3.2% |
| North Central Bronx Endocrinology | 54.5% | 85.8% | 31.3% | 88.5 | 67.8 | -20.7 | 9.5% | 31.1% | 21.6% |
| North Central Bronx Nephrology | 77.6% | 84.7% | 7.1% | 73.1 | 50.0 | -23.1 | 35.2% | 42.8% | 7.6% |
| North Central Bronx Urology | 34.9% | 65.1% | 30.2% | 65.0 | 56.5 | -8.4 | 10.6% | 23.4% | 12.8% |
| Woodhull Urology | 82.6% | 92.9% | 10.3% | 47.6 | 54.3 | 6.7 | 38.1% | 33.6% | -4.6% |

© 2021 Gaye et al. *JAMA Health Forum.*
